# Dr James Robineau Margolis – One of the “Originals”4/10/1943 — 1/6/2023

**DOI:** 10.1016/j.jscai.2023.100593

**Published:** 2023-02-02

**Authors:** Jeffrey W. Moses

**Affiliations:** Division of Cardiology, Columbia University Irving Medical Center, New York, New York

James (Jim) Margolis, one of the world pioneers of percutaneous coronary intervention (PCI), passed away this month after a prolonged illness. Jim dedicated his life to his 2 passions: interventional cardiology and family. Jim was one of the trailblazers of our field and his legacy is present every day in our interventional practices and in the lives of countless patients who have benefited from his work.

After graduating from the University of Illinois College of Medicine in 1968, he served as a research associate at the National Heart and Lung Institute, Framingham, Massachusetts. He completed his training at Duke University Medical Center in Durham, North Carolina, and served as the director of cardiovascular follow-up medicine from 1974 to 1975. In 1976 he moved to Miami and was appointed the director of the cardiovascular laboratory until 1991 and the director of cardiovascular laboratory, Miami Heart Institute, from 1991 to 1997. He held the appointment of clinical professor of medicine at the University of Miami since 1979 and was the member of clinical advisory board of Advanced Cardiovascular Systems since 1988.

In 1977 he became fascinated with the reports of Andreas Gruentzig’s first percutaneous transluminal coronary angioplasty (PTCA), and at the age of 35 he rushed to Zurich to participate in Gruentzig’s earliest demonstration courses. He returned to the US with the “originals”: Simon Stertzer, Richard Myler, David O. Williams, and others and launched the original National Heart, Lung, and Blood Institute (NHLBI) PTCA registry. He thus was amongst the first physicians in the world to perform the procedure. He was an ardent innovator and believer in expansion of the technology in new directions. His creativity and talent led him to be *the* major force in PCI in Florida (indeed south of Atlanta) for a generation. Always on the cutting edge, he was one of the first to perform chronic total occlusion (CTO) PCI, and of course, there were his famous “Ramboplasties.” His hunger for innovation led to many industry collaborations over the years, leading to seminal breakthroughs in intravascular ultrasound tissue characterization and renal denervation, among many others.

Perhaps his most lasting legacy was his Snowmass interventional meeting that ran from 1985 to 2017. Combining his 2 great passions—innovation in PCI and skiing—this unique format brought together worldwide leaders in PCI from the 4 corners of the Earth. This forum allowed for international exchange of science and techniques from Asia, South America, Europe, and North America in an open, collaborative venue. Some of the earliest reports of breakthrough technologies were presented here in a workshop format. It spawned comradery and endless numbers of scientific collaborations among the worldwide leaders in percutaneous vascular therapies for years. Of course, Jim was always in the front row taking notes and asking endless questions, but the highlight of the meeting was being invited to ski with the “Kamikazes”…then you knew you had arrived.

While he loved the outdoors, golfing, and photography, his greatest dedication was to his family and his innumerable friends all around the world. Always embracing and warm, his meeting became a family. He is survived by his wife M. Pauliina Margolis, daughter Susan Gachman, son Michael Margolis, stepson Niklas Ramo, stepdaughter and son-in-law Sophie and Paul Tucker, and his grandchildren Lexi and Jordan Gachman.

In lieu of flowers, a contribution to Parkinson’s disease research is appreciated.

He will be greatly missed.

